# Mutational analysis of compound heterozygous mutation p.Q6X/p.H232R in *SRD5A2* causing 46,XY disorder of sex development

**DOI:** 10.1186/s13052-022-01243-4

**Published:** 2022-03-24

**Authors:** Liwei Li, Junhong Zhang, Qing Li, Li Qiao, Pengcheng Li, Yi Cui, Shujun Li, Shirui Hao, Tongqian Wu, Lili Liu, Jianmin Yin, Pingsheng Hu, Xiaowei Dou, Shuping Li, Hui Yang

**Affiliations:** 1 The Clinical Laboratory, Xingtai People’s Hospital, Xingtai, China; 2 Department of Pathology, the Affiliated Hospital of Guizhou Medical University, Guiyang, China; 3 Department of Orthopaedics, the Affiliated Hospital of Guizhou Medical University, Guiyang, China; 4 Clinical Research Center, the Affiliated Hospital of Guizhou Medical University, Guiyang, China; 5 Department of Burn and Plastic Surgery, the 8th medical center of Chinese PLA General Hospital, Beijing, China; 6 Department of Ultrasound, Xingtai People’s Hospital, Xingtai, China

**Keywords:** 46,XY DSD, *SRD5A2*, p.Q6X/p.H232R mutation, Dihydrotestosterone, 5α-reductase 2 catalytic efficiency

## Abstract

**Background:**

Over 100 mutations in the *SRD5A2* gene have been identified in subjects with 46,XY disorder of sex development (DSD). Exploration of *SRD5A2* mutations and elucidation of the molecular mechanisms behind their effects should reveal the functions of the domains of the 5α-reductase 2 enzyme and identify the cause of 46,XY DSD. Previously, we reported a novel compound heterozygous p.Q6X/p.H232R mutation of the *SRD5A2* gene in a case with 46,XY DSD. Whether the compound heterozygous p.Q6X/p.H232R mutation in this gene causes 46,XY DSD requires further exploration.

**Methods:**

The two 46,XY DSD cases were identified and sequenced. In order to identify the source of the compound heterozygous p.Q6X/p.H232R mutation, the parents, maternal grandparents, and maternal uncle were sequenced. Since p.Q6X mutation is a nonsense mutation, p.H232R mutation was transfected into HEK293 cells and dihydrotestosterone (DHT) production were analyzed by liquid chromatography–mass spectrometry (LC–MS) for 5α-reductase 2 enzyme activities test. Apparent michaelis constant (Km) were measured of p.H232R mutation to analyze the binding ability change of 5α-reductase 2 enzyme with testosterone (T) or NADPH.

**Results:**

The sequence results showed that the two 46,XY DSD cases were the compound heterozygous p.Q6X/p.H232R mutation, of which the heterozygous p.Q6X mutation originating from maternal family and heterozygous p.H232R mutation originating from the paternal family. The function analysis confirmed that p.H232R variant decreased the DHT production by LC–MS test. The Km analysis demonstrated that p.H232R mutation affected the binding of *SRD5A2* with T or NADPH.

**Conclusions:**

Our findings confirmed that the compound heterozygous p.Q6X/p.H232R mutation in the *SRD5A2* gene is the cause of 46,XY DSD. p.H232R mutation reduced DHT production while attenuating the catalytic efficiency of the 5α-reductase 2 enzyme.

**Supplementary Information:**

The online version contains supplementary material available at 10.1186/s13052-022-01243-4.

## Background

46,XY disorder of sex development (DSD) is characterized by incomplete gonadal development and a female or ambiguous phenotype in individuals with a normal 46,XY karyotype [1]. The main clinical phenotypes of 46,XY DSD include bilateral undescended testes and female external genitalia, but without uterus or ovaries [2–4]. The predicted morbidity among subjects with 46,XY DSD is up to 1:20,000. Among affected cases, the morbidity of hypospadias and cryptorchidism can reach 1:200 to 1:300 [5, 6]. Many etiological factors can give rise to 46,XY DSD, with the common causes including androgen insensitivity syndrome (AIS) [7–9] and steroid 5α-reductase type 2 deficiency [10, 11]. AIS is an X-linked recessive genetic disorder that is caused by androgen receptor dysfunction [3]. 5α-Reductase type 2 deficiency is an autosomal recessive disorder caused by decrease or loss of 5α-reductase type 2 function [12].

5α-Reductase type 2, which is coded by the *SRD5A2* gene, is mainly expressed in epididymides, seminal vesicles, prostate, and genital skin [13]. The function of 5α-reductase type 2 is to convert Tto DHT, which is responsible for male sexual development [14, 15]. The loss of function of 5α-reductase type 2 leads to a failure of normal differentiation into the external genitalia, urethra, and prostate and causes 5α-reductase type 2 deficiency. To date, over 100 mutations of the *SRD5A2* gene have been identified [16].

Recently, we identified a compound heterozygous mutation (p.H232R/p.Q6X) of the *SRD5A2* gene in two cases from a single family diagnosed with 46,XY DSD [17]. DNA sequencing showed that the heterozygous p.H232R mutation in the *SRD5A2* gene originated from the paternal side of the family and the heterozygous p.Q6X mutation in the *SRD5A2* gene originated from the maternal side. To elucidate the effect of the p.H232R mutant on the activity of the 5α-reductase type 2 enzyme, HEK293 cells were transfected with p.H232R mutant. LC–MS showed that the mutation clearly decreased DHT production, indicating that this mutation reduced catalytic efficiency. Hence, our results confirmed compound heterozygous mutation (p.Q6X/p.H232R) as the cause of the 46,XY DSD and that p.H232R mutation decreased the catalytic efficiency of the 5α-reductase type 2 enzyme.

## Results

### Clinical phenotype of 46,XY DSD patients

**Fig. 1:**
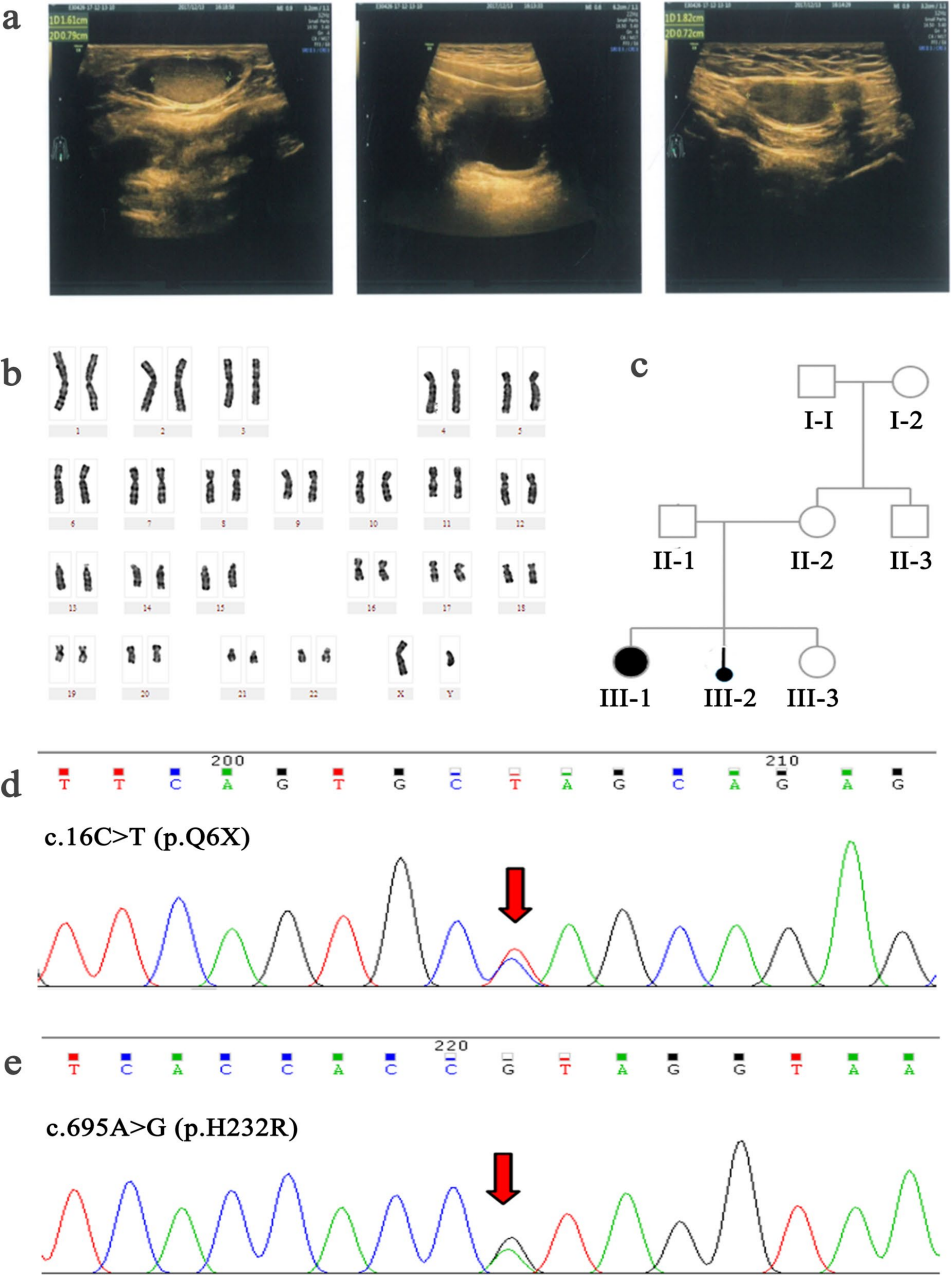
Clinical features of the case 1 (III-1). **a.** B-ultrasound analysis of the case’s internal reproductive organs showed the absence of a uterus and ovaries, but testes on the left labia and right groin. **b.** Karyotype analysis revealed that the case’s karyotype was 46,XY. **c.** Pedigree of the case’s family; Males, females and the patient are indicated by squares, circles, filled circle, respectively. the case 1 (III-1) and her sister (case 2, III-2) were 46,XY DSD patients, while the other relatives did not suffer from 46,XY DSD. **d.** Sequencing analysis of the *SRD5A2* gene. The heterozygous mutation 16C > T found in the patient lead to a stop codon. **e.** Sequencing analysis of the *SRD5A2* gene. The heterozygous mutation 695A > G was found, causing amino acid 232 to change from histidine to arginine. Red arrows indicate mutated nucleotide

Case 1 (III-1): We previously reported a 2-year-old female diagnosed with 46,XY DSD. Clinical examination showed that this proband had normal female external genitalia, but with a blind-ending vagina. An ultrasound test revealed that she had testis in the left labium majus pudendi and right groin, but did not have a uterus or ovaries (Fig. 1a). Karyotype analysis indicated that the proband had a 46,XY karyotype, with no apparent anomalies in chromosome number or structure (Fig. 1b). The proband showed lower levels of follicle-stimulating hormone (FSH), luteinizing hormone (LH), and T (< 0.1 ng/ml) than normal males (Table 1). Family members of the proband were also examined and a pedigree was generated. Two members of this family were diagnosed with 46,XY DSD (Fig. 1c). The sequencing results showed that case 1(III-1) having 46,XY DSD was compound heterozygous for a previously reported nonsense mutation p.Q6X (c.16C > T) (Fig. 1d) [17] and a novel missense *SRD5A2* mutation p.H232R (c.695A > G) (Fig. 1e).

**Table 1:** Characteristics of sex hormones of the case1 (III-1)

Sex Hormone	Mean	Normal Male Range
FSH (mIU/ml)	0.54	1.27–19.26
LH (mIU/ml)	0.12	1.24–8.62
E2 (pg/ml)	27	20–47
PROG (ng/ml)_	0.35	0.1–0.84
T (ng/mL)	< 0.1	1.75–7.81
PRL ( ng/ml)	10.74 ng/mL	2.64–13.3

Serum levels of FSH, LH, E2, PROG, T, and PRL of the case 1 (III-1) were measured by radioimmunoassay

**Fig. 2:**
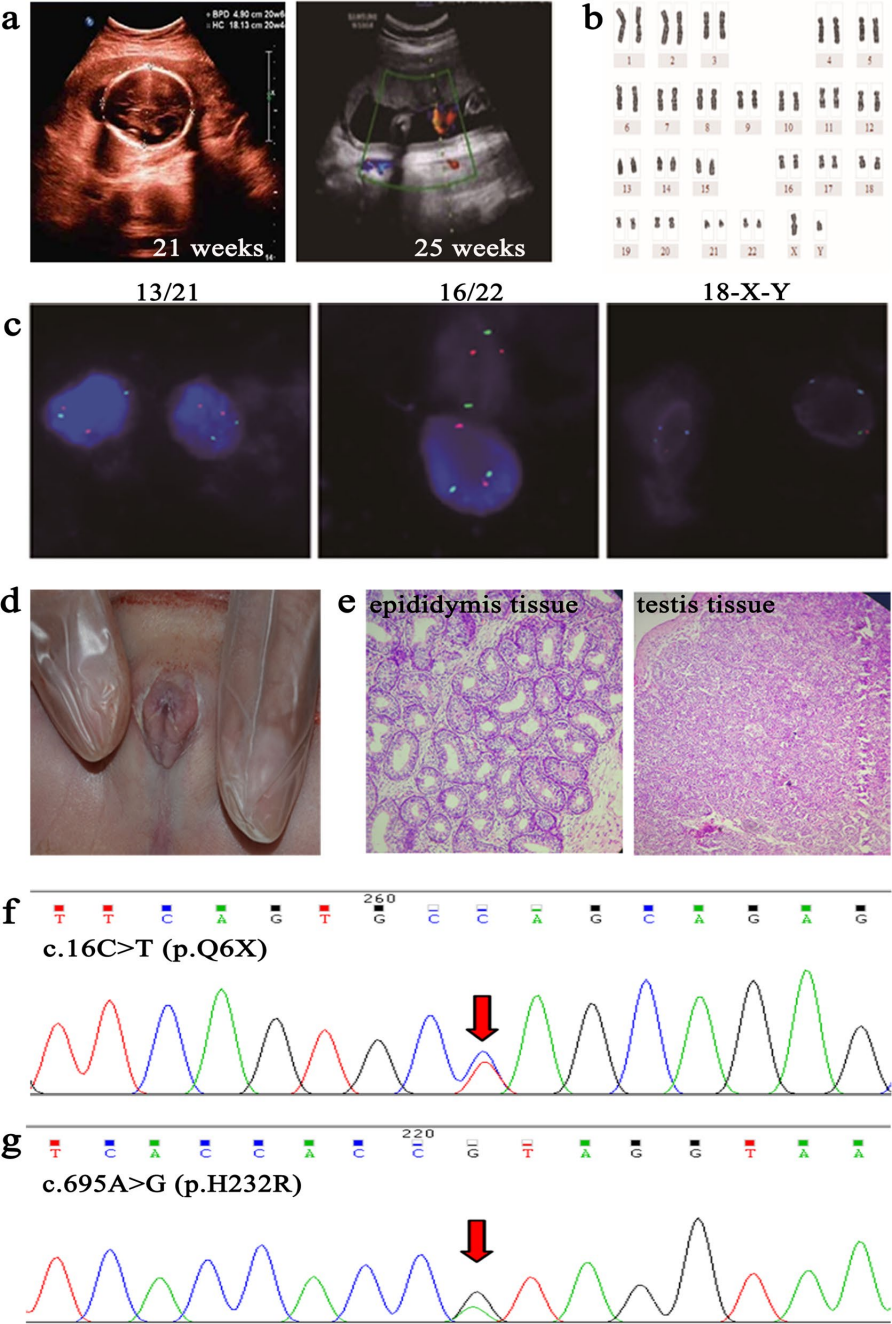
Clinical features of the case 2 (III-2). **a.** Prenatal B-ultrasound testing of the case 2 at 21 and 25 weeks revealed no abnormalities. **b.** Chromosome karyotype analysis showed a normal 46, XY karyotype. **c.** Fluorescence in situ hybridization (FISH) prenatally diagnosed 13/16/18/21/22/X/Y chromosomes in the case 2. **d.** The physical examination of the fetus aborted at 25 weeks’ gestation displayed female external genitalia, but with blind ending vagina. **e.** HE staining showed the fetus had epididymis tissue and testis tissue. **f.** Sequencing analysis of the *SRD5A2* gene and the p.Q6X (c.16C > T) mutation were found. **g.** Sequencing analysis of the *SRD5A2* gene and the p.H232R (c.695A > G) mutation were found. Red arrows indicate mutated nucleotide

Case 2 (III-2): At 21 weeks’ and 25 weeks’ gestation, case 1’s mother came to our hospital for a B-ultrasound examination. The results showed that the fetus did not have male external genitalia (Fig. 2a). Cytogenetic analysis revealed that the child had a 46,XY karyotype, with no apparent anomalies in chromosome number or structure (Fig. 2b). The results of FISH assay to test chromosomes 13/16/18/21/22/X/Y revealed that the child had normal X and Y chromosomes (Fig. 2c). These results indicated that the fetus had 46,XY DSD. Considering the fetus may face difficulty of being unable to treat after birth, the mother decided to terminate the pregnancy. According to the appearance, we found that the external genitalia of the fetus is not a normal male genitalia(Fig. 2d). However, through histopathological examination of the external genitalia, the epididymis and testicular tissues were found (Fig. 2e). Sequencing analysis was performed and showed that Case 2 (III-2) also had the nonsense *SRD5A2* mutation p.Q6X (Fig. 2f) and the novel missense *SRD5A2* mutation p.H232R (Fig. 2g).

### Genetic analysis identified the sources of *SRD5A2* mutations

**Fig. 3:**
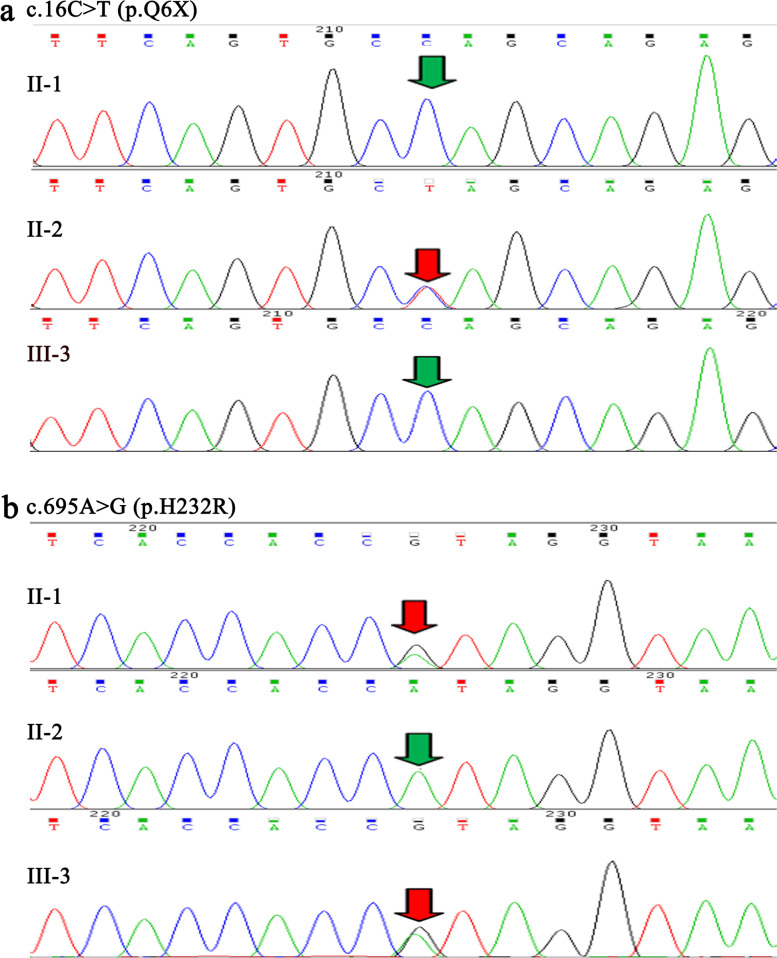
Sequence analysis of *SRD5A2* in the cases’ father II-1, mother II-2 and younger sister III-3. **a.** The heterozygous mutation p.Q6X (c.16C > T) in *SRD5A2* gene was only found in their mother, not found in their father and younger sister. **b.** The heterozygous mutation p.H232R (c.695A > G) in *SRD5A2* gene was found in their patient's father and younger sister, not found in their mother. Red arrows indicate mutated nucleotide. Green arrows indicate unmutated nucleotide

To find the familial sources of the *SRD5A2* gene mutations, Sanger sequencing was performed in six members of the cases’ family. The sequencing results showed that cases’ father II-1 and younger sister III-3 had no the *SRD5A2* mutation p.Q6X, while their mother II-2 had (Fig. 3a). The cases’ father II-1 and younger sister III-3 had the *SRD5A2* mutation p.H232R, but their mother II-2 had no (Fig. 3b). These results demonstrated that the missense mutation p.Q6X of 46,XY DSD cases was derived from the maternal side of the family, while the novel *SRD5A2* mutation p.H232R was from the paternal side.

**Fig. 4:**
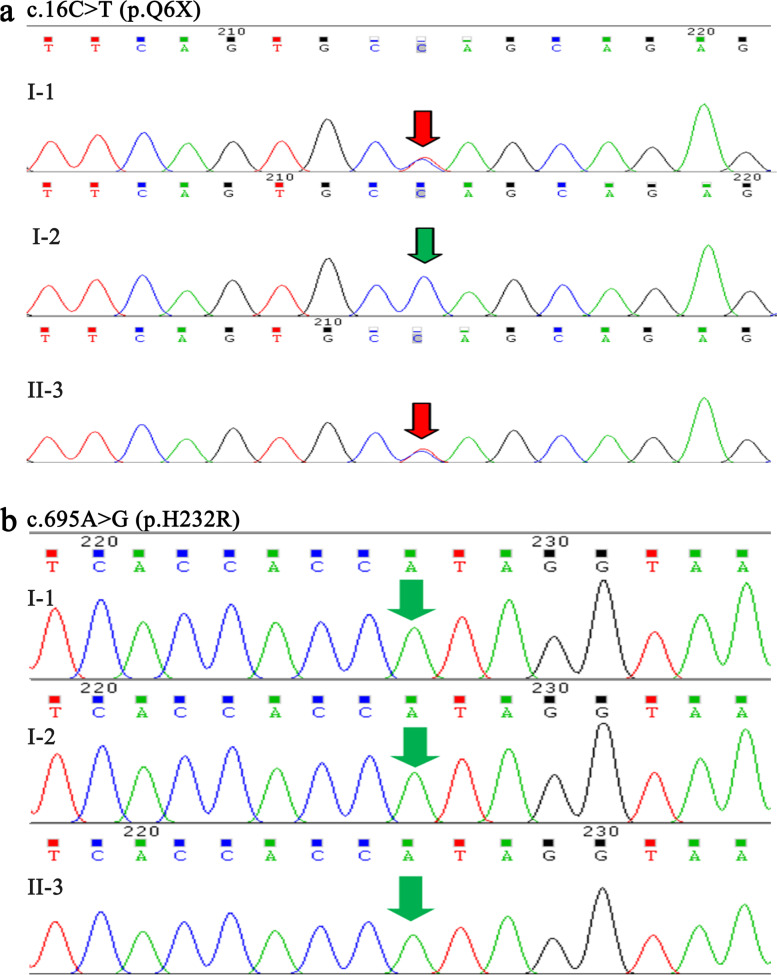
Sequencing analysis of *SRD5A2* gene in the cases’ grandfather I-1, grandmother I-2 and maternal uncle II-3. **a.** The heterozygous mutation p.Q6X (c.16C > T) in *SRD5A2* gene was found in the cases’ grandfather and maternal uncle, but not found in their grandmother. **b.** The heterozygous mutation p.H232R (c.695A > G) in *SRD5A2* gene was not found in the cases’ grandfather, grandmother and maternal uncle. Red arrows: mutated nucleotides. Green arrows: unmutated nucleotides

To further search for the sources of the mutation p.Q6X/p.H232R, Sanger sequencing was performed on cases’ maternal uncle II-3, grandfather I-1, and grandmother I-2. As expected, the maternal uncle II-3 and grandfather I-1 had the nonsense mutation p.Q6X (Fig. 4a), but not the novel mutation p.H232R (Fig. 4b). Cases’ grandmother had neither the mutation p.Q6X (Fig. 4a) nor the mutation p.H232R (Fig. 4b). These results confirmed that the nonsense *SRD5A2* mutation p.Q6X originated from cases’ maternal grandfather pedigree.

Pedigree analysis clearly demonstrated that the *SRD5A2* mutations exhibited autosomal recessive inheritance. Neither the novel *SRD5A2* mutation p.H232R nor the nonsense mutation p.Q6X alone caused 46,XY DSD to develop, but their co-occurrence gave rise to this condition.

### p.H232R mutation reduced the catalytic efficiency of the 5α-reductase type 2 enzyme

**Fig. 5:**
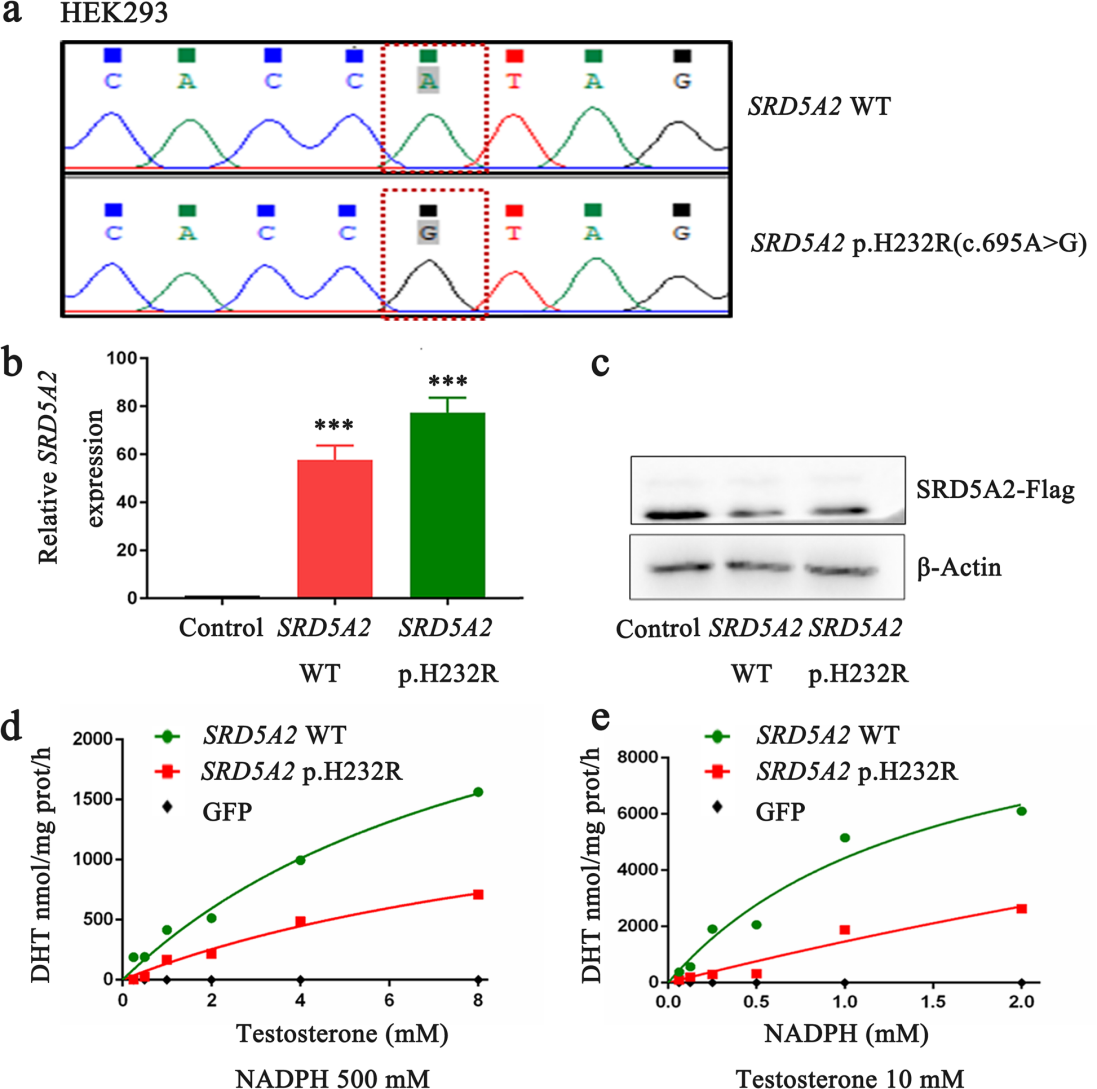
Validation of *SRD5A2* wild-type (WT) and p.H232R mutant HEK293 cell models. **a.** The HEK293 cells were transiently transfected with WT or p.H232R (c.695A > G) mutant *SRD5A2* plasmids. Sequencing analysis showed the mutation c.695A > G in *SRD5A2* in HEK-293 cells transfected with p.H232R mutant plasmids, causing amino acid 232 to change from histidine to arginine. **b.** The transcription of the *SRD5A2* gene in HEK-293 cells transfected with WT and p.H232R mutant plasmids using qRT-PCR. The β-actin gene was used as an internal control. The amounts of *SRD5A2* transcripts were calculated by the standard 2 − ΔΔCt method and were made into a histogram. **c.** Western blot results of *SRD5A2* protein in HEK-293 cells transfected with WT-Flag and p.H232R-Flag mutant plasmids. β-Actin was used as an internal control. **d.** Enzyme activity analysis of *SRD5A2* WT and mutants H232R binding with T by LC–MS. **e.** Enzyme activity analysis of *SRD5A2* WT and mutants H232R binding with NADPH by LC–MS

To investigate the effect of p.H232R mutation on the activity of the enzyme encoded by the *SRD5A2* gene, we transfected wild-type *SRD5A2*, p.H232R mutant *SRD5A2*, or control pcDNA3.1-GFP plasmids into HEK293 cells. DNA sequencing confirmed that p.H232R mutation was present in the cells transfected with p.H232R mutant *SRD5A2* (Fig. 5a). qPCR and western blot confirmed that wild-type or p.H232R mutant *SRD5A2* transfection upregulated the *SRD5A2* expression in HEK293 cells (Fig. 5b, c). LC–MS analysis showed that p.H232R mutation in the *SRD5A2* gene obviously decreased DHT production when cultured with various concentration of testosterone. (Fig. 5d). Using GraphPad Prism 8.0, we found a Km of 12.5 μM, maximum velocity of enzyme-catalyzed reaction (Vm) of 1843 nmol DHT/mg protein/h, and catalytic efficiency (Vmax/Km) of 147.4 nmol DHT/mg protein/h/(μmol/L). In contrast, the cells transfected with plasmid vectors expressing wild-type *SRD5A2* had a Km of 9.3 μM, Vmax of 3352 nmol DHT/mg protein/h, and catalytic efficiency (Vmax/Km) of 360.4 nmol DHT/mg protein/h/(μmol/L). These results indicated that p.H232R mutation affected the binding of *SRD5A2* with T. In addition, with various concentration of NADPH, LC–MS analysis confirmed that p.H232R mutation decreases the catalytic activity of 5α-reductase 2 (Fig. 5e). Using GraphPad Prism 8.0, we found that the p.H232R mutant enzyme had a Km 11.3 μM, a Vm of 18,038 nmol DHT/mg protein/h, and a catalytic efficiency of 1596.3 nmol DHT/mg protein/h/(μmol/L). In contrast, in the wild-type *SRD5A2*, the enzyme activity possessed a Km of 1.5 μM, a Vm of 11,077 nmol DHT/mg protein/h, and the catalytic efficiency was 7384.67 nmol DHT/mg protein/h/(μmol/L). These resluts showed that p.H232R mutation affected the binding of *SRD5A2* with NADPH. Our mutagenesis studies demonstrated that p.H232R mutation reduced the 5α-reductase 2 enzymatic activity via disrupting the binding of *SRD5A2* with T or NADPH.

### Conservation analysis of *SRD5A2* in humans and other species

**Fig. 6:**
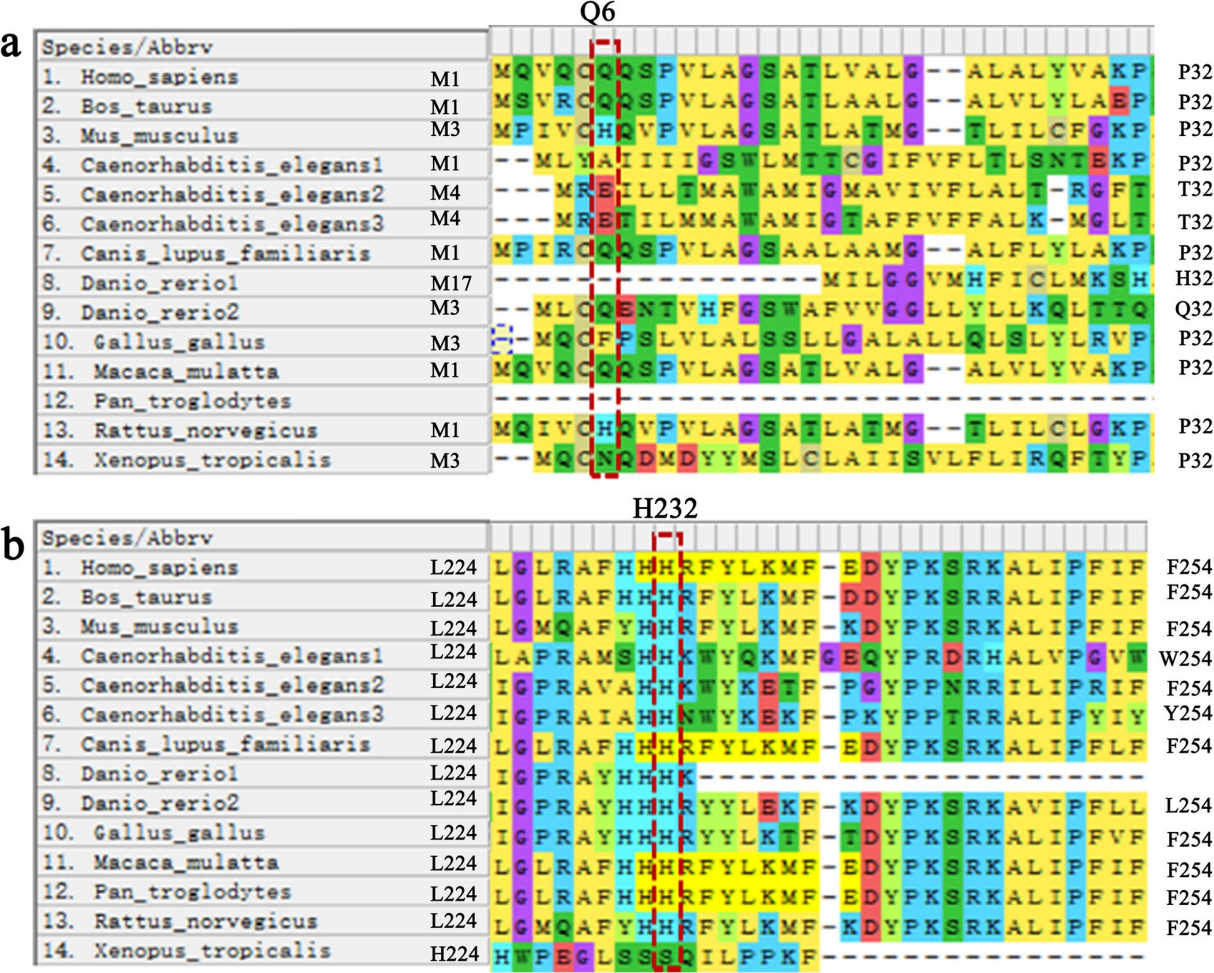
Conservation of the mutation site.** a** Conservation analysis of *SRD5A2* p.Q6X (c.16C > T). **b.** Conservation analysis of *SRD5A2* p.H232R (c.695A > G)

We used MEGA software to analyze the conservation of residues 6 (Fig. 6a) and 232 (Fig. 6b) (marked by red boxes) of the *SRD5A2* gene. The H232 amino acid was found to be highly conserved among different species, including human, mouse, rat, cow, nematode, zebrafish, chicken, macaque, chimpanzee, and clawed frog. This suggested that the H232 amino acid is important in organisms and that mutation at this site has a major effect on gene expression.

### In silico prediction of pathogenicity of human *SRD5A2* p.H232R mutation

Because the identified mutation p.H232R is novel and its pathogenicity is unknown, we first predicted its pathogenicity using software. Three widely used software tools for in silico prediction were used to predict the effect of the human *SRD5A2* p.H232R variant: Polyphen2 [18] score was 1, the SIFT [19] score was 0.007, and the PROVEAN [20] score was − 7.330. These scores indicated that the human *SRD5A2* p.H232R mutation is highly likely to be pathogenic (for details, see Table 2).

**Table 2:** Prediction of the pathogenicity of *SRD5A2* p.H232R mutation

Gene	Nucleotide substitution	Amino acid substitution	Software			
			**Polyphen2**	**SIFT**	**PROVEAN**	
*SRD5A2*	c.695A > G	p.H232R	1	0.007		− 7.330

The pathogenicity score of each software tool is presented. PolyPhen2 software begins with a score of 0.0. Scores in the range of 0.447–0.909 indicate possibly damaging, while scores of 0.910–1.000 are probably damaging. SIFT score < 0.05 was considered to indicate that the mutation affects protein function and ≥ 0.05 was considered to indicate that it was tolerated. The PROVEAN score threshold was − 2.5, with a deleterious effect being considered with a score of ≤  − 2.5 and a neutral effect with a score of >  − 2.5

## Discussion

In this study, we first found that two 46,XY DSD patients in a single family had a compound *SRD5A2* mutation (p.H232R/p.Q6X) [17]. Further sequencing within this family demonstrated that the novel *SRD5A2* p.H232R mutation or the nonsense p.Q6X mutation alone would not result in the development of 46,XY DSD. LC–MS showed that the novel mutation (p.H232R) clearly decreased DHT production and *SRD5A2* catalytic efficiency.

Mutations in the *SRD5A2* gene are common genetic defects causative of 46,XY DSD, which include point mutations, deletions, and insertions [21]. *SRD5A2* functions in converting T into DHT [22]. During early development, DHT is responsible for the formation of the male external genitalia, urethra, and prostate [23, 24]. Some mutations in the *SRD5A2* gene cause 5α-reductase-2 deficiency syndrome [12]. In one Thai patient with male pseudohermaphroditism, sequencing showed the compound mutation p.Q6X/p.G203S in the *SRD5A2* gene [25]. Moreover, in one Chinese patient with hypospadias, sequencing revealed the compound mutation p.G203S/p.Q6X in the *SRD5A2* gene [26]. In our research, the two 46,XY DSD cases had the compound heterozygous *SRD5A2* mutation p.H232R/p.Q6X. The findings showed that the heterozygous *SRD5A2* mutation (p.H232R) or the heterozygous *SRD5A2* mutation (p.Q6X) alone would not give rise to 46,XY DSD. Our and others’ researches support that the compound heterozygous *SRD5A2* mutation (p.H232R/p.Q6X) results in 46,XY DSD.

The *SRD5A*2 gene, located on chromosome 2p23.1, encodes a 254-amino-acid protein and contains five exons and four introns [24]. The p.Q6X mutation in the *SRD5A2* gene, reported only in Asians, creates a drastically truncated protein with complete loss of enzymatic activity due to the lack of both the T- and the NADPH-binding domains [26, 27]. Histidine 232 is located in a stretch of three histidines (residues 230–232) in the *SRD5A2* enzyme. H230P mutation in the *SRD5A2* protein inactivates its enzyme activity [28, 29]. Meanwhile, H231R mutation in the *SRD5A2* protein impairs its isozyme activity since the mutation primarily affects the ability of the enzyme to bind T [29, 30]. Therefore, we speculate that H232R mutation in the *SRD5A2* protein could also inactivate or impair *SRD5A2* enzyme activity. Taking this previous work together with our findings, the compound heterozygous *SRD5A2* mutation p.H232R/p.Q6X could inhibit DHT formation and cause 46,XY DSD.

Most identified *SRD5A2* mutations can reduce DHT levels and cause deficiency of 5α-reductase, which is an autosomal recessive disorder [31, 32]. The p.H232R mutation, located in exon 4 of *SRD5A2*, is in the T-binding domain and most likely affects the binding of 5α-reductase 2 to T, rendering the former less active. Indeed, it was reported that p.H231R mutation in exon 4 of the *SRD5A2* gene causes 46,XY DSD via a mechanism associated with impaired binding of T [30]. Because amino acids 230, 231 and 232 are located in exon 4 and involve the same mutation of histidine to proline or arginine, the mutational impacts are likely to be similar, both preventing binding of the enzyme to its substrate or coenzyme [28, 29]. Our analysis of the enzymatic activity of the *SRD5A2* p.H232R mutation showed that the Vmax/Km value was lower than that of WT *SRD5A2* and reduced the *SRD5A2* catalytic efficiency, suggesting that the p.H232R mutation contributes to the development of genital ambiguity.

## Conclusions

Our research strongly suggested that the compound heterozygous *SRD5A2* mutation p.H232R/p.Q6X leads to 46,XY DSD and revealed the effect of the *SRD5A2* p.H232R mutation on *SRD5A2* catalytic efficiency. Further research is needed to construct a mouse model of 46,XY DSD with the *SRD5A2* p.H232R mutation, replicate the 46,XY DSD disease phenotype, and investigate the regulatory mechanism of p.H232R on steroid 5α-reductase 2 activity in vivo.

## Methods

### Patients

Case 1, a 2-year-old girl with 46,XY DSD, attended our hospital. Physical examination of the female external genitalia was performed. B-ultrasound was carried out to detect male or female internal genital organs, including testis, uterus, and ovaries. In addition, hormone assay and karyotype analysis were performed, along with the sequencing of 219 DSD-related genes.

Case 2, case 1’s younger sibling, was suspected of having 46,XY DSD at 25 weeks’ gestation. Type B ultrasonic test, karyotype analysis, and *SRD5A2* gene sequencing for 46,XY DSD were performed. Upon abortion of the fetus, physical examinations were performed for female external genitalia and HE staining for testis tissue and epididymis tissue.

Written informed consent for the genetic studies was obtained from the family, and all analyses were approved by the Medical Ethics Committee of the People’s Hospital of Xingtai City.

### Hormone assays

Serum levels of FSH, LH, estradiol (E2), progestin (PROG), T, and prolactin (PRL) of the case 1 (III-1) were measured by radioimmunoassay.

### Karyotype analysis

Peripheral blood cells from the proband were cultured in RPMI-1640 medium supplemented with 100 U/ml penicillin, 100 µg/ml streptomycin, and 10% fetal bovine serum (FBS) for 72 h. Colchicine (20 μg/ml) was added for 2 h to arrest cells in metaphase and inhibit spindle body formation. Samples were incubated for 20 min with 75 mM potassium chloride to spread out the spindle body and fixed in Carnoy’s solution. The fixed cells were dropped onto glass slides and placed in an incubator at 75 °C for 3 h to air-dry. Giemsa solution was used to stain the G-bands of the chromosomes.

### FISH analysis

The uncultured amniotic fluid cells from the aborted sibling of the case 1 (III-1) were obtained for FISH analysis. The probes were used to test chromosomes 13, 16, 18, 21, 22, X, and Y.

### Genetic analysis

Peripheral blood samples of case 1, her aborted sibling, surviving sibling, father, mother, maternal uncle, grandfather, and grandmother were obtained for DNA sequencing. Genomic DNA was extracted using the TIANamp Blood DNA Kit (Tiangen), in accordance with the manufacturer’s instructions. Sequencing of 219 of the case 1 (III-1)’s DSD-related genes was performed by BGI using Illumina Genome Analyzer IIx (Supplementary Table 1). The mutated genes in case 1(III-1) and her relatives as listed above were subjected to Sanger sequencing. Mutations were identified by comparing the sequencing results of the case to the UCSC reference genome using the BWA tool.

### Human *SRD5A2* site-directed mutagenesis

Human *SRD5A2* cDNA was kindly provided by Professor Jiahuai Han (Xiamen University) and was subcloned into vector pcDNA3.1 (with 3Flag tag, purchased from Life Technologies). To generate 695A > G mutation in the *SRD5A2* gene, the pcDNA3.1-*SRD5A2* plasmid was used as a template and the QuikChange II Site-Directed Mutagenesis Kit (Catalog #200,523; Agilent) was used to produce the mutation site, in accordance with the manufacturer’s instructions. The primers for cloning the 695A > G mutation in the *SRD5A2* gene were as follows: SRD5A2 mF-695: gcgagcttttcaccaccGtaggttctacctcaagatgtttg, and SRD5A2 mR-695: catcttgaggtagaacctaCggtggtgaaaagctcgcag.

### Transfection assay

HEK293 cells (purchased from the Chinese Academy of Sciences Cell Bank in Shanghai) were cultured in Dulbecco’s Modified Eagle’s Medium supplemented with 10% fetal bovine serum (Gibco) and 1% streptomycin/penicillin (Gibco). The cells were transiently transfected with 2.0 μg of wild-type or p.H232R mutant *SRD5A2* plasmids in each well of a six-well plate using TurboFect™ Transfection Reagent (Thermo Scientific), in accordance with the manufacturer’s protocol. The transfected HEK293 cells were cultured in 5% CO_2_ at 37 °C for 48 h before the assays.

### DNA sequencing of transfection plasmids

HEK293 cells were transfected with wild-type or p.H232R mutant *SRD5A2* plasmids for 48 h and Sanger sequencing was used to assess the DNA sequence. Briefly, the DNA samples were isolated by TIANamp Genomic DNA kit (Cat# DP304-02; TIANGEN, China). The concentration and purity of the DNA were determined by NanoDrop (at 260/280 nm, ND2000C; Thermo). The DNA samples were amplified by polymerase chain reaction (PCR) and sequenced by Genewiz Corporation (Suzhou, China). Primer sequences for the PCR were as follows: SRD5A2-Fseq: AGCCCGTTAAGCAGTTGAGG, and SRD5A2-Rseq: CGGCTTCTTCCGCTTCTTGA.

### Quantitative real-time PCR

Quantitative real-time PCR (qRT-PCR) was performed to assess the mRNA expression after transfection of the wild-type or p.H232R mutant *SRD5A2* plasmid for 48 h. Total RNA was isolated by RNAiso Plus (Cat# 9109; TaKaRa) and reverse transcription was performed using the PrimeScript™ RT Reagent Kit (RR047A; Takara), in accordance with the manufacturer’s protocol. qPCR was performed using PrimeScript™ RT Reagent Kit (RR037A; Takara) with the ViiA7 Real-time PCR System (ABI). The PCR schedule was as follows: 95°C for 30 s, followed by 40 cycles of 95°C for 5 s and 60°C for 34 s. For the relative quantification of *SRD5A2* mRNA, the 2^−ΔΔCt^ method was performed. The primer sequences for qPCR were as follows: SRD5A2F: GCCACTTTGGTCGCCCTT, SRD5A2R: CTCCGTGTGCTTCCCGTAG, β-actinF: AGAGCTACGAGCTGCCTGAC, and β-actinR: AGCACTGTGTTGGCGTACAG.

### Western blot

Western blot was performed as previously described [33]. Briefly, after HEK293 cells has been transfected with wild-type or p.H232R mutant *SRD5A2* plasmids for 48 h, they were collected from six-well plates and lysed with RIPA buffer (P0013; Beyotime Biotechnology). The whole-protein lysates were separated by 12% SDS-PAGE and then transferred to nitrocellulose membranes. After blocking with 5% milk, the membranes were incubated with the primary antibodies monoclonal anti-flag (F9291; Sigma) and β-actin (sc-69879; Santa) at 4°C overnight. After rinsing with Tris-buffered saline containing 1% Tween-20 (TBST), the membrane was then incubated with appropriate HRP-conjugated secondary antibodies (sc516102; Santa Cruz) and detected with an ECL Plus kit (P1050; Applygen).

### Kinetic assays

Kinetic assays were performed to assess the influence of p.H232R variant on the binding of 5α-reductase 2 enzyme to testosterone or NADPH. The HEK293 cells transfected with control vector GFP, *SRD5A2* or *SRD5A2* pH232R was cultured with 500 μl of fresh medium containing 500 μM NADPH (CAS: 100929–71-3, N302057; Aladdin) and various concentrations of T (0.25–8.0 μmol/L, CAS: 58–18-4, M163044; Aladdin). Or the HEK293 cells transfected with control vector GFP, *SRD5A2* or *SRD5A2* pH232R was cultured with 500 μl of fresh medium containing 10 μM T and various concentrations of NADPH (0.0625–2 mM/L). After incubation for 4 h, the medium was collected. The steroids were extracted with chloroform, condensed by a freeze-drying apparatus, and re-dissolved in chromatographic methanol forLC-MS analysis, using buffer A containing 1 mM formate (F112034; Aladdin) and buffer B containing 100% methanol. DHT was quantified according to the concentration and peak area of the standard DHT [CDCT-C10255010; ANPEL Laboratory Technologies (Shanghai) Inc.]. The protein concentration was determined using BCA assays (P0012S; Beyotime). The rate of enzyme production (nmol/mg protein/h) was calculated as an indicator of enzyme activity. The data were processed by Prism8 (GraphPad). The experiments on each group were repeated three times. The data are presented as mean ± SEM.

### Sequence alignment of *SRD5A2* in humans and other species

FASTA format *SRD5A2* sequences of homologous species were downloaded from the NCBI database. Protein sequences of *SRD5A2* were aligned between humans and the other homologous species using MEGA software.

### Analysis of the pathogenicity of human *SRD5A2* p.H232R mutation

The pathogenicity of the human SRD5A2 p.H232R mutation was analyzed using the following bioinformatic programs: Polyphen-2 (http://genetics.bwh.harvard.edu/pph2/), SIFT (http://sift.jcvi.org), and PROVEAN (http://provean.jcvi.org/seq_submit.php).

## Supplementary Information

**Additional file 1. **

## Data Availability

The data that support the findings of this study are available on request from the corresponding author.

## Abbreviations

DSD: Disorder of sex development
DHT: Dihydrotestosterone
T: Testosterone
LC–MS: Liquid chromatography–mass spectrometry
Km: Apparent michaelis constant
AIS: Androgen insensitivity syndrome
FSH: Follicle-stimulating hormone
LH: Luteinizing hormone
Vm: Maximum velocity of enzyme-catalyzed reaction
E2: Estradiol
PROG: Progestin
PRL: Prolactin
