# Supplementary material for: Mutational analysis of compound heterozygous mutation p.Q6X/p.H232R in SRD5A2 causing 46,XY disorder of sex development
Source: Ital J Pediatr. 2022 Mar 24;48:47. doi: 10.1186/s13052-022-01243-4 (PMC8944008; doi:10.1186/s13052-022-01243-4)
Supplement: Supplementary file 1 — Additional file 1. [file 13052_2022_1243_MOESM1_ESM.docx]

**Supplementary table 1:** Test of 219 disorders of sex development-related genes

| **Diseases** | | **Related Genes** |
| --- | --- | --- |
| Sex  Chromosomal Abnormality | | *SRY, BMP15, FGD1, KDM5C, GK, NR0B1, ARX, SMS, MBTPS2, HCCS, OFD1, MID1, KAL1, AR, OPHN1, MED12, HDAC8, ATRX, BRWD3, POF1B, CUL4B, OCRL, PHF6, IGSF1, SOX3, MAMLD1, MECP2, NAA10, FLNA, NSDHL, PCDH11Y, DDX3Y, USP9Y, DGKK, KDM5D, EIF1AY, GATA1, EBP, MTM1, KLHL4, DIAPH2, USP26, HPRT1, FMR1, MTCP1, BRCC3* |
| Germinal Aplasia | | *SRY, NR0B1, NR5A1, CBX2, MAP3K1, DHH, AKR1C2, AKR1C4, DMRT1, DMRT2, DMRT3, DOCK8, SMARCA2, SOX9, SOX3, WNT4, WT1, ARX, ATRX, GATA4, CFTR, CYP19A1, POR, CYP11B1, CYP17A1, HSD3B2, CYP11A1, STAR, AMH, AMHR2, AR, MAMLD1, SRD5A2, LHCGR, HSD17B3, DHCR7, NR3C1, POF1B, FOXL2, BMP15, NOBOX, FIGLA, FSHR, PSMC3IP, INSL3, RXFP2* |
| Others | *TAC3, TACR3, GNRH1, KISS1, WDR11, HS6ST1, SEMA3A, KAL1, FGFR1, PROKR2, PROK2, CHD7, FGF8, GNRHR, KISS1R, NSMF, LHB, NR0B1, LEP, LEPR, FSHB, PCSK1, BBS10, TRIM32, BBS12, MKS1, CEP290, TMEM67, WDPCP, BBS1, BBS2, ARL6, BBS4, BBS5, MKKS, BBS7, TTC8, BBS9, PEX3, PEX14, PEX1, PEX5, PEX12, PEX6, PEX2, PEX26, MAP2K1, PTPN11, KRAS, SOS1, RAF1, NRAS, BRAF, ROR2, WNT5A, EVC, EVC2, FRAS1, FREM2, HSD17B4, HARS2, CUL4B, OPHN1, KDM5C, RAB40AL, SMS, BRWD3, IRF6, RIPK4, EIF2B1, EIF2B2, EIF2B3, EIF2B4, EIF2B5, PAX2, RET, UPK3A, H6PD, HSD11B1, AGPAT2, BSCL2, SOX2, BMP4, HCCS, STRA6, SEMA3E, CUL7, FGD1, KIF7, ALMS1, FGFR2, PITX2, BLM, PHF6, CD96, RAB23, NSDHL, ERCC8, INSR, CHRM3, TP63, ICK, NOTCH2, IRX5, MBTPS2, CDKN1C, PMM2, UBR1, MLL2, GHR, OCRL, LMNA, RAB3GAP2, ORC1, NBN, NAA10, GPC6, MID1, MED12, FLNA, GLI3, SOX10, STK11, MECP2, RECQL4, CREBBP, TBCE, ATR, OFD1, TBX3, HOXD13, HDAC8, DCAF17, BUB1B, SLC39A4, WNT7A, PAPSS2, ALX4, GK, CYB5A, ZBTB16, IGSF1, GNAS, POLG, SF3B4, CFTR, POLR3A, HOXA13, TSHR, WNT3, HFE, TSPYL1, RSPO1, PRKAR1A, BMPR1B, ARID1B, INPP5E, AIRE, SLC29A3* | |
